# Does the Addition of Tramadol and Ketamine to Ropivacaine Prolong the Axillary Brachial Plexus Block?

**DOI:** 10.1155/2014/686287

**Published:** 2014-05-06

**Authors:** Ahmet Can Senel, Ozlem Ukinc, Alper Timurkaynak

**Affiliations:** ^1^Department of Anesthesiology, Medical Faculty of Karadeniz Technical University, 61080 Trabzon, Turkey; ^2^Department of Orthopedics, Medical Faculty of Karadeniz Technical University, 61080 Trabzon, Turkey

## Abstract

*Background and Objectives*. A prospective, randomized, controlled, double-blind clinical trial to assess the effect of tramadol and ketamine, 50 mg, added to ropivacaine in brachial plexus anesthesia. *Methods*. Thirty-six ASA physical statuses I and II patients, between 18 and 60 years of age, scheduled for forearm and hand surgery under axillary brachial plexus block, were allocated to 3 groups. Group R received 0.375% ropivacaine in 40 mL, group RT received 0.375% ropivacaine in 40 mL with 50 mg tramadol, and group RK received 0.375% ropivacaine in 40 mL with 50 mg ketamine for axillary brachial plexus block. The onset times and the duration of sensory and motor blocks, duration of analgesia, hemodynamic parameters, and adverse events (nausea, vomiting, and feeling uncomfortable) were recorded. *Results*. The onset time of sensorial block was the fastest in ropivacaine + tramadol group. Duration of sensorial and motor block was the shortest in the ropivacaine + tramadol group. Duration of analgesia was significantly longer in ropivacaine + tramadol group. *Conclusion*. We conclude that when added to brachial plexus analgesia at a dose of 50 mg, tramadol extends the onset and duration time of the block and improves the quality of postoperative analgesia without any side effects.

## 1. Introduction

Regional anesthesia provides a safe anesthesia for upper extremity surgery. Brachial plexus block with axillary approach for hand and forearm surgery is commonly used.

The use of adjuvants in combination with local anesthetics for peripheral nerve blocks enhances the quality and duration of anesthesia and postoperative analgesia. Numerous studies have been published on the effects of different adjuvants on local anesthetics for axillary brachial plexus block [1–5]. Tramadol and ketamine are the most common adjuvants used with local anesthetics [6, 7].

Tramadol is a synthetic analgesic drug that is antagonized by *α*_2_-adrenoceptor antagonists as well as opioid antagonists [8]. Ketamine, a dissociative anesthetic N-methyl-D-aspartate (NMDA) antagonist, abolishes peripheral afferent noxious stimulation [9].

The effects of different doses of tramadol, ranging between 40 and 200 mg, and ketamine, ranging between 1 and 1.5 mg/kg, with different local anesthetics, have been reported in several studies [10–13]. However, there is no study that addresses the minimal dose required to prolong the duration of motor block, sensorial block, and analgesia without increasing adverse effects.

We designed a prospective, randomized, controlled, double-blind clinical trial to assess the effect of lower doses of tramadol and ketamine, 50 mg, added to ropivacaine in brachial plexus anesthesia to determine effectivity of tramadol and ketamine.

## 2. Methods

After institutional ethics committee approval and written informed consent were obtained, 36 ASA physical statuses I and II patients, between 18 and 60 years of age, scheduled for forearm and hand surgery under axillary brachial plexus block, were included in this randomized (envelope method), controlled study. Pregnant women and patients with a history of cardiac, respiratory, hepatic, or renal failure were excluded from the study. A 20-gauge intravenous cannula was inserted into the contralateral dorsal hand. Routine monitoring of electrocardiogram, noninvasive measurement of arterial blood pressure, peripheral oxygen saturation, and respiratory rate monitoring were conducted.

Patients were allocated to 3 groups in a controlled, randomized, double-blinded fashion. Group R received 0.375% ropivacaine in 40 mL, group RT received 0.375% ropivacaine in 40 mL with tramadol 50 mg, and group RK received 0.375% ropivacaine in 40 mL with ketamine 50 mg for axillary brachial plexus block.

Patients were premedicated with fentanyl 0.75 *μ*g/kg and midazolam 0.03 mg/kg intravenously 10 minutes before the axillary block. Axillary block was performed in the supine position with the upper arm abducted at 90° and the elbow flexed at 90°. The area was shaved the day before and disinfected. The axillary artery was palpated in the proximal part of the axilla, and a skin wheal was injected using 1 mL of lidocaine 2%. A nerve stimulator (Stimuplex Kanule A 50, B Braun, Melsungen, Germany) was used to identify the plexus. The position of the needle was judged adequate when an output current of less than 0.5 mA still elicited a slight distal motor response. With intermittent aspiration, the total volume was injected into the perivascular area. All the blocks were performed by the same anesthetist.

The onset times and the duration of sensory and motor blocks, duration of analgesia, hemodynamic parameters, and adverse events (nausea, vomiting, and feeling uncomfortable) were recorded. Patients were considered sedated according to Ramsay Sedation Scale > 2 (Table 1).

All local anesthetic solutions and adjuvant drugs were prepared by an anesthesiologist not involved in performing brachial plexus block or data collection. All blocks were performed by one of the authors, who was unaware of the contents of the injected solution. Sensory block and motor block of musculocutaneous, radial, median, and ulnar nerves were assessed and recorded at 5-minute intervals. Sensory block of each nerve was assessed by pinprick and compared with the same stimulation of the contralateral arm as reference. Motor block was evaluated by modified Bromage scale (0 = no motion, 1 = finger movement, 2 = wrist flexion, and 3 = elbow flexion). Duration of sensory block was considered as the time interval between the local-anesthetic administration and the complete offset of anesthesia. Motor-block duration was defined as the time interval between the local-anesthetic administration and the recovery of motor function. Heart rate, peripheral oxygen saturation, respiratory rate, and blood pressure were measured before the axillary block, 5, 10, 15, 30, 45, and 60 minutes after the axillary block, and every 60 minutes thereafter for 2 hours postoperatively. Additional adverse events were recorded (bradycardia, dizziness, nausea, vomiting, and sedation).

Statistical analyses of the data were performed using *t*-tests and analyses of variance for parametric data to compare the differences between groups. We used Mann-Whitney *U* tests for nonparametric comparison of the data. A value of *P* < 0.05 was considered statistically significant. The results are expressed as means ± standard errors.

## 3. Results

There was no statistically significant difference between the groups in age, weight, gender, and duration of surgery (Table 2). No differences in the quality of sensory and motor blocks before and during the surgery were noted among the groups; none of the patients required supplemental analgesic during surgery. Systolic, diastolic, and mean arterial pressures were not significantly different between groups (*P* ≥ 0.05). Also, heart rates and oxygen saturations of all of the groups were not significantly different (*P* ≥ 0.05).

The distribution of the surgery types among groups is shown in Table 3, and there were no differences in the number of hand, forearm, or elbow surgeries in the groups. The onset and duration of motor block and sensorial block are shown in Table 4.

The onset time of sensorial block was the fastest in ropivacaine + tramadol group, 8.17 ± 0.33 min, compared to the ropivacaine, 9.55 ± 0.34 min (*P* = 0.015), and ropivacaine+ketamine groups, 9.85 ± 0.41 (*P* = 0.007). The onset time of motor block was 10.8 ± 0.38 min, 9.3 ± 0.28 min, and 11.1 ± 0.43 min, respectively, and *P* values were *P* = 0.005 and *P* = 0.002.

Duration of sensorial block was also the shortest in the ropivacaine + tramadol group, 13.16 ± 0.40 h, compared to the ropivacaine, 14.90 ± 0.33 h (*P* = 0.023), and ropivacaine + ketamine groups, 13.6 ± 0.30 h (*P* = 0.009). Duration of motor block was similar, in groups 15.8 ± 041 h, 13.60 ± 0.30 h, and 13.74 ± 0.33 h, respectively, and *P* values were *P* = 0.001 and *P* = 0.001.

Duration of analgesia was 21.6 ± 0.40 h in group R, 24.9 ± 0.33 h in group RT (*P* = 0.001), and 22.6 ± 0.30 h in group RK compared with group R (*P* = 0.08).

There was no complication during the blocking of brachial plexus blocking. We found no statistically significant differences with regard to side effects between the ropivacaine and ropivacaine + tramadol groups, but side effects were more frequently recorded in the ropivacaine and ketamine groups. Six patients in ropivacaine+ketamine group felt uncomfortable; five reported dizziness and eight reported nausea, while only two patients reported nausea in the other groups. Two patients were considered sedated in groups R and RT, but six were considered sedated in group RK (Table 5).

## 4. Discussion

The results of this study suggest that the addition of 50 mg tramadol to 0.375% ropivacaine for axillary brachial plexus block prolongs the duration of anesthesia and analgesia without increasing side effects, whereas addition of 50 mg ketamine to 0.375% ropivacaine does not provide any additional effect.

There are few studies about the addition of tramadol to local anesthetics in axillary brachial plexus block. Most published experience has been obtained with the dose of 100 mg of tramadol with different local anesthetics.

Kapral et al. assessed the use of tramadol with the 1% local anesthetic mepivacaine. They concluded that 100 mg tramadol significantly prolongs the motor and sensorial block of brachial plexus block without any side effects [14].

In a study by Sarihasan et al., use of 100 mg tramadol as an adjuvant to bupivacaine in supraclavicular plexus block improved the quality of anesthesia and extended the duration of postoperative analgesia [15].

The study of Robaux et al. also demonstrates that 40, 100, and 200 mg tramadol added to 1.5% mepivacaine for brachial plexus anesthesia extends the duration and improves the quality of postoperative analgesia in a dose dependent fashion with acceptable side effects [10]. Therefore, direct comparison of our study to these three studies is of limited value.

In some studies, 100 mg tramadol was used in different ropivacaine volumes. Kesimci et al. added 100 mg of tramadol to 40 mL of ropivacaine 7.5 mg/mL and observed a longer duration of analgesia but did not observe an improved speed of onset of block or an increase in the duration of sensory or motor block [16]. In the only study in which tramadol was added to 20 mL of 7.5 mg/mL ropivacaine, by Antonicci, it was demonstrated that tramadol significantly reduced the onset time of brachial plexus block and prolonged the duration of anesthesia and postoperative analgesia [17]. Kesimci et al. reported that the cause of the discrepancy between their findings and the findings of Antanucci may be the higher dose of (150 mg) injectate used at the same concentration of ropivacaine.

Unlike the aforementioned studies, our study used 50 mg tramadol as an adjuvant to 40 mL 0.375% ropivacaine to determine if this minimal dose of tramadol speeds the onset time of the sensorial and motor block and prolongs the duration of axillary brachial plexus block compared to ropivacaine alone. In our study addition of 50 mg tramadol supports the results of Antonicci rather than Kesimci with longer duration of analgesia only without prolonging onset and the duration of sensory or motor block. However, direct comparison to our study is of limited value because different local anesthetics with different volumes and concentrations were used.

Ketamine was another adjuvant used in this study that was reported as an effective agent with local anesthetics. The contributory effect of the addition of ketamine, an N-methyl-D-aspartate (NMDA) antagonist, was evaluated when the drug is delivered via caudal [6, 8], epidural [18, 19], and spinal [20, 21] routes. In all these studies, addition of ketamine with antagonism to NMDA receptors and an axonal conduction block may also contribute to the analgesic mechanism of regional ketamine to different local anesthetics which provided a success on both block duration and quality. Senel et al. reported that 50 mg ketamine, as an adjuvant to local anesthetic, is an effective dose in spinal anesthesia. In the study of Lee et al., the addition of 30 mg ketamine in a volume of 30 mL ropivacaine 0.5% did not improve the onset and duration of brachial plexus block because of a low dose of ketamine; however, there was a relatively high incidence of adverse effects [22].

In our study, addition of 50 mg ketamine to 0.375% ropivacaine did not provide any additional effect on axillary brachial plexus. The possible cause of this may be the low concentration of 50 mg ketamine in a volume of 40 mL. In our opinion, a higher concentration of ketamine is necessary for ketamine to be used as an adjuvant to local anesthetics in brachial plexus block. However, using a higher concentration means more ketamine-induced adverse effects.

Few studies report adverse effects when various doses of tramadol and ketamine are added to local anesthetics as adjuvants. These studies found no difference in adverse effects when comparing the ropivacaine alone group to a group in which 100 mg tramadol was added for brachial plexus. Our study confirmed these results [23–25].

We found no statistically significant differences with regard to the side effects between the groups R and RT, but all side effects in the RK group, except dizziness and sedation, were significantly different.

Nausea and vomiting were observed in the RK group, possibly due to the emetic effect of ketamine that is caused by release of endogenous catecholamine. Feelings of discomfort are also known effects of ketamine.

We conclude that when added to brachial plexus analgesia at a dose of 50 mg, tramadol extends the onset and duration time of the block and improves the quality of postoperative analgesia without any side effects. Further studies of the effects of lower doses of tramadol with various combinations of tramadol and local anesthetics are needed, before final recommendations can be made.

Our findings do not encourage the use of ketamine for brachial plexus block. Ketamine does not enhance the local anesthetic effect at the level of the axillary brachial plexus nerve roots and does not prolong postoperative analgesia with the dose of 50 mg when combined with ropivacaine. In addition, the high incidence of ketamine-induced adverse effects at this dose is disturbing.

These findings make us ask the question of “do we need to use ketamine as an adjuvant to local anesthetics in brachial plexus blockage?”

The answer is that we have more than one alternative.

## Figures and Tables

**Table 1:** Ramsay sedation scale.

Score	Response
1	Anxious or restless or both
2	Cooperative, orientated, and tranquil
3	Responding to commands
4	Brisk response to stimulus
5	Sluggish response to stimulus
6	No response to stimulus

**Table 2:** Characteristics of the patients.

	Group R *n* = 12	Group RT *n* = 12	Group RK *n* = 12
Age (yr)	34.30 ± 4.61	39.8 ± 2.83	38.5 ± 3.72
Gender (M/F)	10/2	6/6	12/4
Weight (kg)	68 ± 1.8	71 ± 1.4	67 ± 1.0
Duration of surgery (min)	73.8 ± 10.1	65.2 ± 13.6	81.7 ± 9.3
ASA I/II	10/2	9/3	10/2

Note: values are mean ± SD.

**Table 3:** Types of surgery.

Types of surgery	Group R *n* = 12	Group RT *n* = 12	Group RK *n* = 12
Tendon reconstruction	3	4	7
Contracture release	2	2	0
Nerve repair	5	2	2
Internal fixation	1	2	2
Mass excision	1	2	1

**Table 4:** Onset and duration of anesthesia and analgesia after axillary block.

	Group R *n* = 12	Group RT *n* = 12	Group RK *n* = 12
Onset of sensorial block (min)	9.55 ± 0.34	8.17 ± 0.33*	9.85 ± 0.41
Onset of motor block (min)	10.8 ± 0.38	9.3 ± 0.28*	11.1 ± 0.43
Duration of sensorial block (h)	13.6 ± 0.40	14.90 ± 0.33*	13.6 ± 0.30
Duration of motor block (h)	13.60 ± 0.30	15.8 ± 0.41*	13.74 ± 0.33
Duration of analgesia (h)	21.6 ± 0.40	24.90 ± 0.33*	22.6 ± 0.30

*Means *P* ≤ 0.05 and statistically significant.

**Table 5:** Side effects between groups.

	Group R *n* = 12	Group RT *n* = 12	Group RK *n* = 12
Emesis	2	2	8*
Vomiting	1	1	6*
Dizziness	3	4	5
Sedation	2	2	6
Feeling uncomfortable	1	2	6*

*Means *P* ≤ 0.05 and statistically significant.
